# Changes in the expression of hippocampal proteins in rats with recrudescence of morphine addiction

**DOI:** 10.3892/etm.2012.861

**Published:** 2012-12-18

**Authors:** HAITANG QIU, YUFENG GAO, YIXIAO FU, LIAN DU, TIAN QIU, KUN FENG, QINGHUA LUO, HUAQING MENG

**Affiliations:** 1 Department of Mental Health, First Affiliated Hospital of Chongqing Medical University, Chongqing 400016;; 2 Second Department, Third Hospital of Zhongshan, Zhongshan 510630;; 3 Department of Mental Health, Yuquan Hospital of Tsinghua University, Beijing 100049, P.R. China

**Keywords:** morphine addiction, hippocampus, proteome, two-dimensional electrophoresis

## Abstract

The high recrudescence rate of drug addiction has received attention worldwide and its mechanisms remain to be elucidated. This study aimed to analyse the disparate protein expression in the hippocampal tissue of rats with recrudescence of morphine addiction, as well as to provide clues for the exploration of the recrudescence mechanism. Sixteen male adult Sprague-Dawley rats were divided equally into the morphine and physiological saline groups. Effective nose pokes were determined as the main index. The proteins were separated using the immobilised pH gradient two-dimensional polyacrylamide gel electrophoresis (2-DE). Disparate protein spots were analysed using the PDQuest 2-DE software. Peptide dactylograms were obtained using the matrix-assisted laser desorption/ionisation time-of-flight mass spectrometry. The effective nose poke counts of the morphine group significantly increased during addiction maturation compared with the saline group (P<0.001). The post-recrudescence nose poke counts of the morphine group significantly increased compared with those before recrudescence (P<0.001). Fifteen disparate proteins were identified according to the protein electrophoresis of the morphine and physiological saline groups, including three proteins associated with energy metabolism, two ionic channel regulatory proteins, one heat shock protein and one exogenous substance metabolic enzyme. The energy metabolism and expression of cell metabolism-related proteins decreased in the hippocampus of rats with morphine recrudescence.

## Introduction

The high recrudescence rate of drug addiction has received attention worldwide and its mechanisms remains to be determined and elucidated.

Drug craving in addiction maturation involves multiple memory circuits, including working, fragmentary and emotional memories (1). In addition, addiction modifies a number of morphological changes in the hippocampus, including organelle reduction, mitochondrial swelling, chromatin margination, karyopyknosis and necrosis, which have been observed in rats with morphine addiction (2,3). Craving extinction is a condition in which an individual with a drug addiction does not present drug craving and seeking behaviour for a certain period after drug withdrawal. However, craving extinction is only an occult state of drug dependency, which is transformed into a recrudescent state through stimulation (4). The recrudescence of drug addiction refers to a condition in which an occult state of drug craving and seeking behaviour turns into an apparent state by ignition, thereby leading to addiction recrudescence (5). The recrudescence of drug addiction is the reinstatement of drug craving following extinction, which is largely associated with learning and memory (6). The hippocampus is the memory centre, an important part of which is the Papez circuit, a key addiction maturation circuit (7).

Effective animal models, including locomotor sensitization, conditioned place preference (CPP), drug discrimination and self-administration models play a crucial role in the study of drug addiction. Locomotor sensitization is easy to perform and has good sensitivity. However, this model does not reflect the subjective desires of the animals involved. CPP is also easy to implement and has a short experimental period; however, it requires a large number of animals and also does not reflect the subjective desires of the animals involved. Drug discrimination is classified as a behavioural test that exhibits the advantages of reflecting the desires of the animals studied, as well as easy animal model maintenance. However, this model has poor sensitivity (5). By contrast, self-administration efficiently reflects the subjective demands of the animal and simulates the process of human addictive behaviour; however, it has a complicated procedure and requires laborious animal maintenance (8,9).

In light of such procedures, intravenous self-administration animal models were established in the present study to investigate the changes in hippocampal protein expression during the recrudescence of morphine addiction. Recrudescence-related specific proteins were then identified. Using these procedures, this study intends to lay a theoretical foundation for research into the molecular biological mechanism of recrudescence and its treatment targets.

## Materials and methods

### Animals

Sixteen adult male, clean-grade Sprague-Dawley rats were supplied by the laboratory animal centre of Zhejiang, China. These animals weighed 240±10 g before the experiment and 220±10 g by the end of the experiment. Specific experimental cages were designed and software devices were programmed according to the SuperState Borland Delphi software. This study was conducted in strict accordance with the recommendations in the Guide for the Care and Use of Laboratory Animals of the National Institutes of Health and the animal use protocol was reviewed and approved by the Institutional Animal Care and Use Committee (IACUC) of the First Affiliated Hospital of Chongqing Medical University.

### Model establishment

Models were established according to the methods used by Weeks (9); however, a number of adaptations were conducted according to the requirements of the current experiment. The experimental procedure was divided into five stages as follows: i) the animals were fed naturally for 3–5 days for acclimation prior to the experiment. Spontaneous nose poke screening tests were carried out 1 day before training. The animals were kept away from food 24 h before the screening. Those rats with a spontaneous nose poke count of >10/4 h during the observation period were excluded. ii) The animals were subjected to a venous cannula treatment of the neck and then allowed to recover for 7 days. During the recovery period, the rats were fed *ad libitum* and received an anti-infection treatment with penicillin for 3 days. iii) The animals were placed into specific cages and were allowed to run for 15 min. They were given water *ad libitum* but were prohibited food during the training. A green light was turned on at the beginning of each training cycle. Whenever an effective nose poke occurred, the light would be turned off. Immediately, the automatic equipment injected morphine (purity 98%, purchased from Zhejiang Provincial Public Security Department, Zhejiang, China) intravenously at 1.0 mg/kg with a pump injection sound. A red light was turned on for 5 sec to indicate an effective nose poke. A 20 sec refractory period ensued after each injection, during which nose pokes were counted but without drug injections administered. The control group was treated under the same method using physiological saline injections at 1.0 mg/kg instead of drug injections. iv) A natural withdrawal method was adopted. The animals were trained for 2 h each day. During the training, the rats were given water *ad libitum* but no food and were placed in an environment without light signals and injections. The training lasted for 12 days. v) The animals were returned to the training cages, in which they were again stimulated by lights and pump injection sounds. Conditions were the same as during the addiction training but without morphine injections. Effective nose poke counts were recorded.

### Hippocampal tissue handling

The animals were decapitated rapidly after anesthetisation. Brain tissue blood was flushed through using pre-chilled physiological saline (4°C). The skull was isolated and the brain tissues were then extracted completely. The brain tissues and fascia on the surface of the hippocampus were removed and placed in an ice bath. The hippocampus was collected, weighed and frozen in liquid nitrogen for 2 min prior to storing at −80°C.

### Tissue protein extraction

The samples in the cryopreservation tube were swirled with pre-chilled deionised water (4°C) and then centrifuged at 3,000 × g for 5 min. The supernatant was removed and 0.1 ml 40 mmol/l Tris was added (Sigma-Aldrich, St. Louis, MO, USA). Each sample was frozen and thawed three times for 1 min. An enzyme was added to obtain the homogenate on ice. The homogenate was allowed to react with the enzyme for 25 min at 4°C. Lysate was added and the homogenate was obtained on ice. After another 25-min reaction period at 4°C, the obtained sample was centrifuged at 13,000 × g for 30 min and the supernatant was collected. The protein concentration was determined using the Bradford method.

### Immobilised pH gradient-based two-dimensional gel electrophoresis (2-DE)

Isoelectric focusing (first dimension) was performed by diluting the sample in heavy rehydration buffer. Rehydration and focusing were conducted automatically at 18°C. The total voltage × working time was ∼75,000 V/h. Then, the following procedure was performed: i) gel strip equilibration. The focused gel strips were equilibrated twice in equilibration buffer and washed in electrophoresis buffer for 1 min.ii) Sodium dodecylsulphate polyacrylamide gel electrophoresis (SDS-PAGE; second dimension) was carried out. iii) Silver nitrate staining. The gels were fixed in stationary liquid overnight, rinsed, sensibilised, washed and kept away from light for 25 min. The gels were washed three times and then stained. Protein expression in the morphine and physiological saline groups were determined repeatedly to obtain stable graphic spectra for the comparison of the protein spots. Repeatability was assessed. iv) Gel image analysis. Gel images were obtained using a GS-800 scanner and Quality One scanning software ChemiDoc XRS (Bio-Rad, Hercules, CA, USA). The intensity correction, spot detection, background subduction, matching and ID correction of the images were performed using PDQuest 2D analysis software (Bio-Rad). Disparate protein spots were obtained by comparing the images of the morphine and physiological saline groups using SPSS software (SPSS Inc., Chicago, IL, USA).

### Mass spectrometry identification

The disparate protein points were cut and digested with trypsin in gel. Peptide fingerprint data were obtained using matrix-assisted laser desorption/ionisation time-of-flight mass spectrometry. Following removal of the interference peaks, the Swiss-Prot database was accessed. The type of rat was limited and an error within 1 Da was allowed. At least four peptides were matched and the formylation and partial oxidation of methionine were allowed. Fifteen disparate proteins were identified.

### Statistical analysis

Data were analysed using SPSS 15.0 software. Effective nose poke counts were presented as the means ± standard error. Paired t-tests were performed for comparisons between groups and one-factor analysis of variance (ANOVA) was used for comparisons within a group during recrudescence. P<0.05 indicated a statistically significant difference.

## Results

### Addiction phase

Compared with the saline group, the morphine group showed a significant difference from 2 days of addiction training (P<0.01). The morphine group arrived at a relatively stable plateau phase of self-administration after 3 days, with a nose poke count of >20/4 h, which was also significantly higher than the saline group (P<0.001). These results are summarised in Table I.

### Extinction phase

A significant difference in the nose poke count between the two groups was observed 2 days before drug withdrawal (P<0.001). After 3 days extinction, the nose poke count in the morphine group dropped below the spontaneous nose poke count (10/4 h). No significant difference in this count was observed compared with the saline group (P>0.05). This state continued until the end of the 12 days of the extinction training. These results are summarised in Table II.

### Recrudescence phase

The effective post-recrudescence nose poke count in the morphine group significantly increased compared with the pre-recrudescence count (P<0.001). Furthermore, the effective pre- and post-recrudescence counts in the morphine group varied significantly from the effective pre- and post-recrudescence counts in the physiological saline group (P<0.001). Although the effective post-recrudescence nose poke count in the physiological saline group also increased compared with the pre-recrudescence count, the difference was not significant. These results are summarised in Table III.

### Distributions of amphitropic protein spots

**Figure 1.**
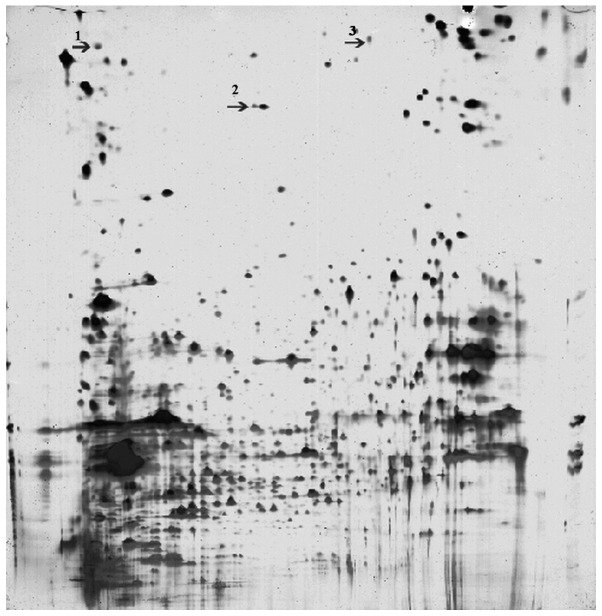
Morphine recrudescence group.

**Figure 2.**
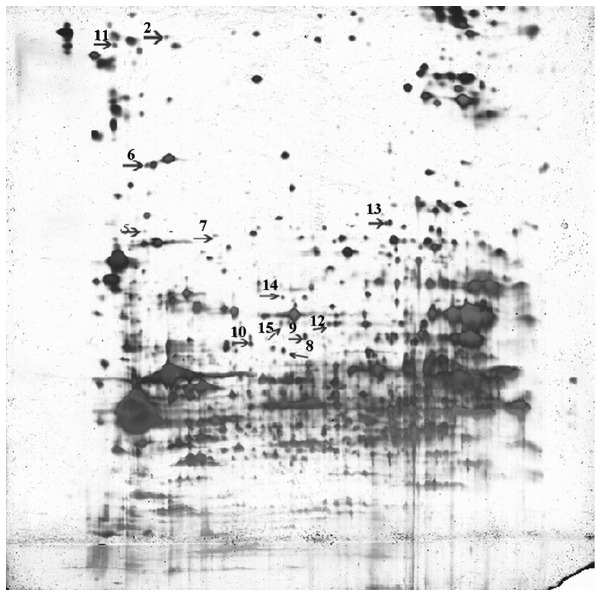
Physiological saline recrudescence group.

The matching rate between the gels of the groups was 76%. The gel images of the two groups were compared using PDQuest software, taking one sheet from each group as a reference. Significant differences in 23 spots were observed (P<0.05; Figs. 1 and 2).

### Disparate protein spots

Fifteen disparate proteins were identified, including three enzymes related to energy metabolism, nucleoside diphosphate (NDP) kinase A, fatty acid-binding protein and nicotinamide adenine dinucleotide (NADH) dehydrogenase (ubiquinone) 1 α subcomplex subunit 10; two ionic channel regulatory proteins, K^+^ channel-interacting protein 2 and mitogen-activated protein kinase 11, as well as carboxylesterase 3, cytoskeleton-associated protein 4 and 14-3-3 protein β/α. These results are summarised in Table IV.

## Discussion

The present study has demonstrated that self-administration models exhibit a relatively stable drug addiction plateau, extinction and recrudescence phases, which satisfies the current primary criteria for recrudescence models (10,11). Thus, this study succeeded in establishing self-administration rat models and simulating the three phases of addiction, maturation, extinction and recrudescence.

Fifteen disparate proteins were identified. Among these proteins, three are associated with energy metabolism, NDP kinase A, fatty acid-binding protein and NADH dehydrogenase (ubiquinone) 1 α subcomplex subunit 10. These proteins participate in the energy supply process through oxidation reduction. The results of this study revealed that their expression significantly decreased in the morphine group during the recrudescence phase. This finding suggests a decrease in hippocampal energy metabolism during the morphine addiction maturation or addiction recrudescence process. NDP kinase A is highly expressed in the sera of stroke patients and therefore serves as a marker for apoplectic seizure. This indicates that its overexpression is a manifestation of an increase in brain function compensation and a stress-induced change due to brain injury (12). NDP kinase A, together with a series of other individual proteins, is linked to performance in the Morris water maze (13). Serotonin 1A receptor knockout mice are commonly used in anxiety and cognitive function tests. NDP kinase A expression decreases in these rat models (14).

Of the disparate proteins identified in this study, two were ionic channel regulatory proteins: K^+^ channel-interacting protein 2 and mitogen-activated protein kinase 11. The former regulates the density of potassium ion receptors in cell membranes and promotes complex formation in the ionic channel (15) and the latter transmits the pressure on the cell membranes and induces gliocytes to activate the p38 mitogen-activated protein kinase channel (16). Thus, the two proteins function in intra and extracellular substance transportation. This study has demonstrated that the expression of K^+^ channel-interacting protein 2 and mitogen-activated protein kinase 11 decreased in the morphine recrudescence group. This finding indicates a decrease in substance transport ability and cell metabolism during the morphine recrudescence phase. Heat shock-related 70 kDa protein 2 is expressed in the hippocampus of normal rats (17). The expression of this protein also significantly decreased in the morphine recrudescence group. Heat shock-related 70 kDa protein 2 functions in the accurate assembly, folding and transport of proteins. It also participates in the maintenance of normal projection transmission and inhibits apoptosis (18). A decrease in its expression may therefore accelerate apoptosis. This finding is in line with the hippocampal atrophy observed following long-term addiction (19).

A decrease in carboxylesterase 3 expression was also observed in the morphine recrudescence group. Carboxylesterase 3 performs a significant role in exogenous material metabolism and specifically binds with morphine in the liver (20). It is also involved in cocaine and morphine metabolism. Therefore, the significant decrease in its expression in the morphine recrudescence group may be closely correlated with the mechanism of recrudescence. In addition, the expression of cytoskeleton-associated protein 4 and 14-3-3 protein β/α decreased in the experimental group, which is consistent with a previous study reporting the decreased expression of these proteins in rats with morphine addiction (20). Presumably, these changes are associated with neural cell reduction and protein fibre demyelination. However, whether the low expression of these two proteins occurs in whole brain tissues or is confined to the hippocampus remains to be explored.

The present study has a number of limitations. Firstly, the changes in brain proteins following addiction are complicated, involving other brain regions, including the hippocampus, nucleus accumbens septum and mesencephalic ventral tegmental area. This study only focused on the hippocampus, thus investigation into these other brain sections should be conducted. Secondly, protein databases are not complete. Consequently, certain proteins may still not be listed. Additionally, the roles of some proteins remain uncertain. Further role verifications for key proteins are therefore necessary.

## Figures and Tables

**Table I.** Effective nose poke counts in the morphine and physio logical saline groups at different time points in the addiction maturation phase.

Day	Morphine group	Physiological saline group
1	7.62±3.41	5.17±2.11
2	13.13±3.31^a^	8.21±2.36
3	21.73±4.05^b^	7.26±3.93
4	27.26±4.63^b^	6.04±1.56
5	22.04±4.55^b^	5.64±1.24
6	25.54±3.66^b^	5.86±1.90
7	25.05±3.14^b^	7.54±3.21
8	27.18±5.17^b^	5.51±1.66
9	28.25±3.83^b^	5.60±1.43
10	31.65±3.37^b^	5.80±1.96
11	30.09±3.55^b^	7.31±2.62
12	34.22±5.61^b^	7.09±2.75
13	31.08±5.14^b^	6.82±1.40

a P<0.01 and

b P<0.001, compared with the physiological saline group on the same day.

**Table II.** Effective nose poke counts in the morphine and physio logical saline groups at different time points in the addiction extinction phase.

Day	Morphine group	Physiological saline group
1	13.60±1.3^b^	6.16±1.62
2	10.71±1.6^b^	4.99±1.26
3	3.72±1.52^a^	2.88±1.12
4	2.23±0.65	2.01±1.03
5	2.41±0.47	2.43±0.84
6	3.04±0.84	2.46±0.56
7	1.08±1.12	1.24±0.63
8	2.09±0.72	0.89±0.39
9	2.12±0.91	1.17±0.36
10	1.32±0.33	0.96±0.45
11	2.02±1.23	2.16±1.17
12	2.11±1.14	1.3±0.9

a P<0.01 and

b P<0.001, compared with the physiological saline group on the same day.

**Table III.** Effective nose poke counts in the morphine and physiological saline groups in the extinction and recrudescence phases.

Phases	Morphine group	Physiological saline group
Extinction	2.6±1.3	2.2±1.1
Recrudescence	26.2±6.2^a,b^	3.2±1.4

a P<0.001, effective nose poke count of the morphine group in the recrudescence phase compared with that in the extinction phase.

b P<0.001, morphine group compared with the physiological saline group in the recrudescence phase.

**Table IV.** Disparate protein spots in the morphine and physiological saline recrudescence groups.

Protein point group	Serial number	Name	Molecular weight/isoelectric point (Da)	Peptides matching	Expression changes with morphine
1	O70139	cAMP-dependent protein kinase inhibitor γ	7944/4.1	5/11	Increase
2	P55051	Fatty acid-binding protein	14864/5.5	11/32	Decrease
3	Q61908	Protein p8 MTCP-1	7743/8.7	4/27	Decrease
4	Q05982	Nucleoside diphosphate kinase A	17193/6.0	4/15	Increase
5	Q9D3N2	EF-hand calcium-binding domain-containing protein 1	24606/4.9	7/23	Decrease
6	Q9D217	Prune homolog 2	20677/4.8	9/69	Decrease
7	Q9JM59	K^+^ channel-interacting protein 2	30993/4.9	6/18	Increase
8	Q561S0	NADH dehydrogenase (ubiquinone) 1 α subcomplex subunit 10	40493/7.6	15/45	Decrease
9	Q8VCT4	Carboxylesterase 3	61788/6.2	7/23	Decrease
10	Q8BMK4	Cytoskeleton-associated protein 4	63693/5.5	10/30	Decrease
11	P51879	Oncomodulin	12260/4.0	4/12	None
12	Q920V68	14-3-3 protein β/α	39371/8.1	17/50	None
13	Q64270	Translation initiation factor eIF-2B subunit α	33679/8.4	5/14	None
14	P14659	Heat shock-related 70 kDa protein 2	69642/5.5	6/27	None
15	Q9WUI1	Mitogen-activated protein kinase 11	41358/5.4	5/22	None

cAMP, cyclic adenosine monophosphate; MTCP, mature T-cell proliferation; NADH, nicotinamide adenine dinucleotide; eIF, eukaryotic initiation factor.
